# Diagnostic value of the electrocardiogram in the assessment of prior myocardial infarction

**DOI:** 10.1007/s12471-020-01515-w

**Published:** 2020-11-16

**Authors:** M. Lobeek, E. Badings, M. Lenssen, R. Uijlings, K. Koster, E. van ’t Riet, F. M. A. C. Martens

**Affiliations:** 1 Department of Cardiology, Deventer Hospital, Deventer, The Netherlands; 2 Department of Radiology, Deventer Hospital, Deventer, The Netherlands; 3 Teaching Hospital Deventer, Deventer Hospital, Deventer, The Netherlands; 4 Department of Cardiology, Isala Hospital, Zwolle, The Netherlands

**Keywords:** Myocardial infarction, Electrocardiogram, Sensitivity, Specificity, Predictive value

## Abstract

**Background:**

The best available imaging technique for the detection of prior myocardial infarction (MI) is cardiac magnetic resonance (CMR) with late gadolinium enhancement (LGE). Although the electrocardiogram (ECG) still plays a major role in the diagnosis of prior MI, the diagnostic value of the ECG remains uncertain. This study evaluates the diagnostic value of the ECG in the assessment of prior MI.

**Methods:**

In this retrospective study, data from electronic patient files were collected of 1033 patients who had undergone CMR with LGE between January 2014 and December 2017. After the exclusion of 59 patients, the data of 974 patients were analysed. Twelve-lead ECGs were blinded and evaluated for signs of prior MI by two cardiologists separately. Disagreement in interpretation was resolved by the judgement of a third cardiologist. Outcomes of CMR with LGE were used as the gold standard.

**Results:**

The sensitivity of the ECG in the detection of MI was 38.0% with a 95% confidence interval (CI) of 31.6–44.8%. The specificity was 86.9% (95% CI 84.4–89.1%). The positive and negative predictive value were 43.6% (95% CI 36.4–50.9%) and 84.0% (95% CI 81.4–86.5%) respectively. In 170 ECGs (17.5%), the two cardiologists disagreed on the presence or absence of MI. Inter-rater variability was moderate (κ 0.51, 95% CI 0.45–0.58, *p* < 0.001).

**Conclusion:**

The ECG has a low diagnostic value in the detection of prior MI. However, if the ECG shows no signs of prior MI, the absence of MI is likely. This study confirms that a history of MI should not be based solely on an ECG.

## What’s new?

- Assessment of the diagnostic value of the electrocardiogram (ECG) in the detection of prior myocardial infarction (MI) compared to the gold standard, cardiac magnetic resonance (CMR) with late gadolinium enhancement (LGE).
- The ECG has a low diagnostic value in the detection of prior MI.
- Future decisions concerning prior MI should not be based solely on an ECG, but should always be substantiated by the data of CMR with LGE.

## Introduction

Despite a decrease in mortality rates, cardiovascular disease (CVD) is still one of the most important causes of death worldwide [1, 2]. In 2019, approximately 38,000 people died from CVD in the Netherlands, 22% died thereof from coronary artery diseases, including myocardial infarction (MI) [3]. To determine strategies for treatment for secondary prevention, it is important to detect prior MI.

Today, the best available imaging technique for the assessment of MI is cardiac magnetic resonance (CMR) with late gadolinium enhancement (LGE) [4, 5]. In acute situations, the electrocardiogram (ECG) has an important role in the diagnostic process [6, 7]. However, only a limited number of studies have addressed the subject of the ECG in the assessment of prior MI compared to CMR with LGE [8, 9]. The diagnostic value of the ECG for the evaluation of prior MI has been questioned [10, 11].

The aim of this study was to assess the diagnostic value of the ECG in detecting prior MI compared to that of the gold standard, CMR with LGE, in a non-academic, medium-sized regional hospital.

## Methods

### Study population

This is a single-centre, retrospective study. All patients who underwent CMR with LGE between January 2014 and December 2017 at the Deventer Hospital were screened for eligibility. Patients were excluded if no ECG was available within 6 months before the CMR or if the electronic medical record (EMR) was not accessible. Finally, a patient was excluded if the outcome of the CMR was inconclusive or if the ECG showed an acute or hyperacute MI.

Data were collected from the EMR by reviewing reports on CMR with LGE, ECGs, letters and reports of clinicians, reports and letters of clinicians in other hospitals, measurements of vital signs, and laboratory results.

### Cardiac magnetic resonance

CMR was performed using a 1.5 T magnetic resonance imaging (MRI) system (Signa HDxt, GE Healthcare, Milwaukee, WI, USA). Scans were carried out using an eight-channel HD cardiac array coil, following the CMR protocol of the hospital. Ten minutes after the administration of 0.2 mmol/kg intravenous gadolinium, LGE images were made, including the two-chamber, four-chamber and short-axis views.

All CMR with LGE images were analysed in a clinical setting (not for research purposes) by an experienced radiologist and discussed by a multidisciplinary team, consisting of a radiologist and at least one cardiologist. CMR images with inconclusive or unclear descriptions in the EMR were reassessed by an experienced radiologist. Patients were divided in two groups: (1) patients with a pattern of LGE typical for MI and (2) those without an LGE pattern typical for MI. A distinction was made between subendocardial hyperenhancement and transmural hyperenhancement (>50% of the myocardium). Images with a pattern of LGE typical for MI were divided in different segments based on the standardised 17-segment model of the American Heart Association [12]. The different segments were merged into two categories: an anterior and inferior category. The anterior category consisted of the segments anterior, anterolateral, anteroseptal, septal and apical; the inferior category consisted of the segments inferior, inferolateral, inferoseptal and lateral.

### *ECG interpretation*

Twelve-lead ECGs were extracted from the EMR and blinded before assessment. ECG assessment was performed by two independent cardiologists separately and analysed as to the presence or absence of signs associated with prior MI without knowledge of the results of CMR with LGE. Both cardiologists used the ECG criteria according to the expert consensus document of the European Society of Cardiology [6]. ECGs with signs of a left or right bundle branch block or signs of ischaemia were marked as negative (absence of MI). Inter-observer disagreement was resolved by the judgement of a third cardiologist.

### Statistical analysis

Statistical analysis was performed using SPSS (version 25, IBM SPSS Statistics for Windows). Continuous data are presented as means with standard deviation or as median with first and third quartile if the data had a non-normal distribution. Analyses were performed using the Student’s *t*-test and Mann-Whitney U test. Categorical data were presented as numbers (*n*) and percentages (%) and compared with the chi-squared test. The diagnostic value of the ECG (sensitivity, specificity, positive predictive value (PPV) and negative predicted value (NPV)) were calculated and shown in a 2 × 2 table. The relationship between the independent variables age, gender, patient history, hypertension, diabetes mellitus (DM), hypercholesterolaemia, (history of) smoking and outcome of the CMR was determined by multivariate logistic regression. Determinants which affected the regression coefficient of the association between ECG classification and MRI outcome by more than 10% were added to the model. Inter-rater variability between the classification of ECGs by the two cardiologists was analysed using Cohen’s kappa [13]. A *p*-value <0.05 was considered to be statistically significant.

The Medical Ethical Committee of the Isala Hospital Zwolle in The Netherlands reviewed the research protocol and concluded that the rules laid down in the Medical Research Involving Human Subjects Act did not apply to this research proposal.

## Results

### Study population

**Fig. 1:**
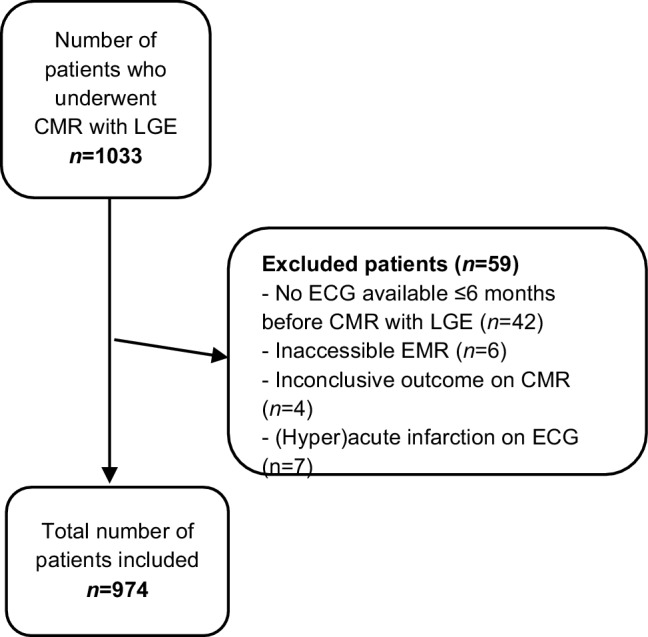
Flowchart of study population. *CMR* cardiac magnetic resonance, *ECG* electrocardiogram, *EMR* electronic medical record, *LGE* late gadolinium enhancement

Of 1033 patients that underwent CMR with LGE between January 2014 and December 2017, 974 were included in the analysis (Fig. 1), 566 men and 408 women. Their median age was 63.5 years. In 205 patients (21.0%) MI was present on CMR with LGE. In this group, the percentage of women (21.0% vs 47.5%, *p* < 0.001) was statistically significantly lower and the median age (67.1 vs 62.3 years, *p* < 0.001) was statistically significantly higher compared to the patient group without MI. An overview of patient characteristics and CMR data is shown in Tab. 1.

**Table 1:** Patient characteristics of the study population and patients with and without myocardial infarction (*MI*) on cardiac magnetic resonance (*CMR*) with late gadolinium enhancement (*LGE*)

	Total	Presence of MI on CMR with LGE		*p*-value
		Yes	No	
*n* (%)	974	205 (21.0%)	769 (79.0%)	–
Age (median (IQR))	63.5 (52.6–71.9)	67.1 (57.1–72.8)	62.3 (51.1–71.4)	<0.001
Women	408 (41.9%)	43 (21.0%)	365 (47.5%)	<0.001
Patient history^a^	167 (17.1%)	103 (50.2%)	64 (8.3%)	<0.001
Diabetes mellitus	147 (15.1%)	56 (27.3%)	91 (11.8%)	<0.001
Hypertension	473 (48.6%)	107 (52.2%)	366 (47.6%)	0.255
Hypercholesterolaemia	337 (34.6%)	96 (46.8%)	241 (31.3%)	<0.001
*Smoking*				
– Ex-smoker	239 (24.5%)	70 (34.1%)	169 (22.0%)	<0.001
– Smoker	246 (25.3%)	71 (34.6%)	175 (22.8%)	<0.001
Family history	192 (19.7%)	43 (21.0%)	149 (19.4%)	0.360
*Pharmaceuticals*				
– Beta blockers	414 (42.5%)	144 (70.2%)	270 (35.1%)	<0.001
– ACEI/ARB	438 (45.0%)	151 (73.7%)	287 (37.3%)	<0.001
– CCB	105 (10.8%)	27 (13.2%)	78 (10.1%)	0.214
– Diuretics	337 (34.6%)	112 (54.6%)	225 (29.3%)	<0.001
– Statins	321 (33.0%)	150 (73.2%)	171 (22.2%)	<0.001
– Insulin	49 (5.0%)	21 (10.2%)	28 (3.6%)	<0.001
– PAI	262 (26.9%)	127 (61.0%)	135 (17.6%)	<0.001
– VKA	78 (8.0%)	34 (16.6%)	44 (5.7%)	<0.001
– NOAC	80 (8.2%)	11 (5.4%)	69 (9.0%)	0.095
– Heparins	20 (2.1%)	12 (5.9%)	8 (1.0%)	<0.001
– Alpha blockers	7 (0.7%)	2 (1.0%)	5 (0.7%)	0.642
– Anti-arrhythmics	43 (4.4%)	14 (6.8%)	29 (3.8%)	0.058
Days between ECG and CMR (median (IQR))	30 (15–42)	21 (6–41)	33 (19–42)	<0.001

*ACEI* angiotensin converting enzyme inhibitor, *ARB* angiotensin II receptor antagonist, *CCB* calcium channel blocker, *ECG* electrocardiogram, *IQR* interquartile range, *NOAC* new oral anticoagulant, *PAI* platelet aggregation inhibitor, *VKA* vitamin K antagonist

^a^Patient history of atherosclerotic disease

### Inter-rater variability

In 170 patients (17.5%) the two cardiologists disagreed in their judgement of the presence or absence of an MI based on the ECG. In 37 of these 170 patients (21.8%), the third cardiologist saw electrocardiographic changes that were associated with a prior MI. There was a moderate inter-rater variability: κ = 0.51 (95% CI 0.45–0.58, *p* < 0.001).

### Diagnostic value

In Tab. 2, the results of ECG assessment and those of CMR with LGE as the gold standard with regard to a history of prior MI are shown. Signs of previous MI based on CMR were seen in 205 patients (21.0%). In 179 out of 974 patients (18.4%) the ECG showed electrocardiographic changes that were associated with a prior MI. The ECG correctly showed signs of a prior MI in 38.0% of the patients with MI on CMR. Of the assessed ECGs, 56.4% were false positive (CMR showed no MI) and 16.0% were false negative. In 84.0% of the patients without MI on CMR, no ECG changes compatible with prior MI were found (Tab. 2).

**Table 2:** Overview of electrocardiogram (*ECG*) and cardiac magnetic resonance (*CMR*) findings compatible with a history of myocardial infarction (*MI*)

		MI on CMR with LGE		
		Yes	No	Total
MI on ECG	Yes	78	101	179
	No	127	668	795
	Total	205	769	974

*LGE* late gadolinium enhancement

The sensitivity of an ECG in detecting an MI was 38.0% (95% CI 31.6–44.8%). The specificity was 86.9% (95% CI 84.4–89.1%). The PPV and NPV of the ECG were 43.6% (95% CI 36.4–50.9%) and 84.0% (95% CI 81.4–86.5%) respectively (Tab. 3).

**Table 3:** Diagnostic value of the electrocardiogram in detecting a prior myocardial infarction compared to cardiac magnetic resonance with late gadolinium enhancement

	%	95% CI
Sensitivity	38.0	31.6–44.8
Specificity	86.9	84.4–89.1
Positive predictive value	43.6	36.4–50.9
Negative predictive value	84.0	81.4–86.5

*CI* confidence interval

**Fig. 2:**
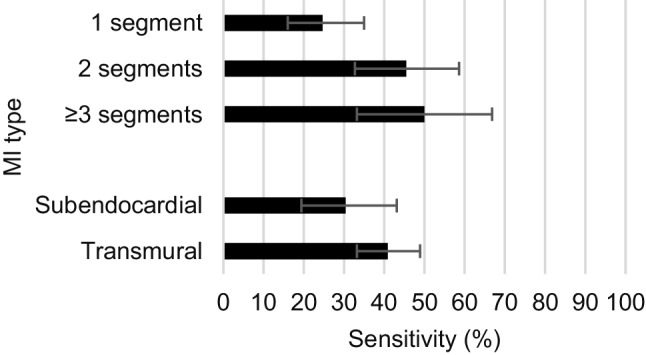
Effect of size and transmurality on the sensitivity of the electrocardiogram in detecting myocardial infarction (*MI*) seen on cardiac magnetic resonance with late gadolinium enhancement; including 95% confidence interval

After categorisation, the ECG showed a sensitivity of 63.3% (95% CI 50.8–74.8%) for an MI in the anterior category, compared to a sensitivity of 20.2% (95% CI 13.1–28.8%) in the inferior category. If MI was seen in both categories, the ECG showed a sensitivity of 43.5% (95% CI 29.8–57.9%). A comparable PPV of the ECG divided into the anterior and inferior category was found (43.0% (95% CI 34.4–51.9%) vs 42.9% (95% CI 30.0–55.9%) respectively). An increase in size and transmurality showed non-significant improvement of the sensitivity (Fig. 2).

The odds ratio of a positive versus a negative ECG for MI on CMR with LGE was 4.062 (95% CI 2.859–5.771). In multivariate logistic regression, a patient history of CVD appeared to be a confounder in the association between ECG findings and MI on CMR with LGE. After correction for this confounder, the odds ratio of a positive versus a negative ECG was 3.394 (95% CI 2.283–5.046). Other patient characteristics (age, gender, presence of DM, hypercholesterolaemia, hypertension and (history of) smoking) were not found to influence the association between ECG findings and MI on CMR with LGE.

## Discussion

In this study, the diagnostic value of the ECG in the diagnosis of a prior MI was assessed compared to that of CMR with LGE. The ECG is a useful tool in the diagnosis of prior MI. After correction for a patient history of CVD, a suspicious ECG increased the likelihood of a prior MI being diagnosed with CMR with LGE, by a factor 3.4. The high specificity of the ECG in the assessment of prior MI (86.9%) showed a high probability of having an ECG without signs of prior MI if no prior MI was seen on CMR with LGE. The high NPV (84.0%) showed that if no signs of prior MI are seen on the ECG, the probability that a patient suffered an MI is low. However, the low sensitivity (38.0%) and PPV (43.6%) of the ECG showed that the ECG alone is not a reliable instrument in the assessment of prior MI. These results confirm the generally accepted opinion that ECG findings should always be interpreted together with symptoms and other diagnostic investigations.

The low diagnostic value of the ECG corresponds with previously reported data. The low sensitivity found in our study also corresponds with the results of previous studies [8, 9, 14–16]. If the ECG was used as a diagnostic tool in the assessment of prior MI, more than 50% of the prior MI would be missed. However, the PPV in this study was lower than that in previously reported studies [8, 9]. The high PPV reported in previous studies might be explained by the higher a priori chance of having an MI. The population in our study resulted in a lower a priori chance, leading to a different PPV value compared to other studies.

### Limitations

The retrospective study design leads to several limitations, including the problem of missing data in the EMR and the inability to check the correctness of certain classifications like family history or other risk factors. Besides, ECGs were classified into two groups: presence or absence of MI on the ECG. However, in clinical practice, there is a wide range of doubtful cases in which the application of predefined criteria can be discussed. This may have influenced the reliability of the results. In clinical practice the physician always has more information, which helps him/her in making a decision, like the presence of risk factors, use of medication, blood tests and previous ECGs. Finally, the external validity of this study is limited. In this study all patients who underwent CMR with LGE between January 2014 and December 2017 were selected. This resulted in a heterogeneous patient group, in which the CMR with LGE was performed for a variety of indications. The results of our study might be different from those in other populations such as patients with a suspicion of CVD.

## Conclusion

In this study, it was demonstrated that it is not feasible to use only the ECG as an instrument in the assessment of prior MI. Future decisions concerning prior MI should not be based solely on an ECG, but should always be substantiated by data from CMR with LGE.

### Recommendations

In future research the above-mentioned limitations could be minimised by using a prospective study design, whereby all patients in whom an ECG is performed also have to undergo CMR with LGE. However, such a study design is not feasible in clinical practice because of the high costs and a lack of availability of CMR systems.

We recommend to at least consider performing CMR with LGE in patients with signs of prior MI on the ECG if the outcome has clinical consequences for secondary prevention.
